# Large-Scale Desynchronization During Interictal Epileptic Discharges Recorded With Intracranial EEG

**DOI:** 10.3389/fneur.2020.529460

**Published:** 2020-12-23

**Authors:** Elie Bou Assi, Younes Zerouali, Manon Robert, Frederic Lesage, Philippe Pouliot, Dang K. Nguyen

**Affiliations:** ^1^University of Montreal Hospital Research Center (CRCHUM), University of Montreal, Montreal, QC, Canada; ^2^Department of Neuroscience, University of Montreal, Montreal, QC, Canada; ^3^Institute of Biomedical Engineering, Polytechnique Montreal, Montreal, QC, Canada; ^4^Montreal Heart Institute, Montreal, QC, Canada

**Keywords:** intracranial electroencephalography, epileptiform discharges, epileptic focus, epilepsy surgery, surgical outcome, functional connectivity

## Abstract

It is increasingly recognized that deep understanding of epileptic seizures requires both localizing and characterizing the functional network of the region where they are initiated, i. e., the epileptic focus. Previous investigations of the epileptogenic focus' functional connectivity have yielded contrasting results, reporting both pathological increases and decreases during resting periods and seizures. In this study, we shifted paradigm to investigate the time course of connectivity in relation to interictal epileptiform discharges. We recruited 35 epileptic patients undergoing intracranial EEG (iEEG) investigation as part of their presurgical evaluation. For each patient, 50 interictal epileptic discharges (IEDs) were marked and iEEG signals were epoched around those markers. Signals were narrow-band filtered and time resolved phase-locking values were computed to track the dynamics of functional connectivity during IEDs. Results show that IEDs are associated with a transient decrease in global functional connectivity, time-locked to the peak of the discharge and specific to the high range of the gamma frequency band. Disruption of the long-range connectivity between the epileptic focus and other brain areas might be an important process for the generation of epileptic activity. Transient desynchronization could be a potential biomarker of the epileptogenic focus since 1) the functional connectivity involving the focus decreases significantly more than the connectivity outside the focus and 2) patients with good surgical outcome appear to have a significantly more disconnected focus than patients with bad outcomes.

## Introduction

### Networks at Work

It has long been recognized that most anatomical subdivisions of the brain exhibit some degree of functional specialization (1, 2), and localization of the neural substrate of brain function has constituted the workhorse of neuroscientists for decades. More recently, it was shown that a deeper understanding of brain functions could be gained by investigating functional specialization at the scale of brain networks. Functional networks emerge as sets of brain regions that exchange information in a spontaneous and transient fashion through synchronization of their activity (3, 4), a process commonly referred to as “functional connectivity.” Importantly, healthy functioning of brain networks relies on precise levels of connectivity among its constituting structures, and alterations of functional connectivity were shown to be involved in various neurological diseases (5).

### Epilepsy as a Network Disease

In epilepsy, it is increasingly recognized that deep understanding of seizures requires both localizing and characterizing the functional network of the region where they are initiated, which is usually referred to as the epileptic focus. As was originally postulated by Spencer, seizures arise when an epileptic focus disrupts the balance of functional connectivity within a network, thereby driving its constituting regions into aberrant discharges (6). This theoretical framework is supported empirically by a number of studies showing permanent alterations in brain connectivity in epilepsy. Indeed, resting-state brain functional connectivity in epileptic patients is significantly different from control subjects (7–9). Interestingly, abnormal increases in functional connectivity seem to be closely associated with evolution of the disease (10) and local hypersynchrony/ hyposynchrony were found to be potential electrophysiological/hemodynamic biomarkers for localizing the epileptic focus (11–15). This clinical perspective was further investigated during epileptiform discharges (16) and seizures (17–19), during which regions with elevated connectivity spatially overlapped with the clinically defined epileptic focus. However, it is still unclear whether functional connectivity increases or decreases at seizure onset, as both findings are reported in the literature. Those contrasting findings cannot be attributed to different recording modalities since contradictions arise both from magnetoencephalography (MEG) (20, 21) and intracranial electroencephalography (iEEG) (22–28) studies. In addition, although those studies evaluate functional connectivity using different metrics, most of them use broad-band cross-correlation and mean phase coherence, which were shown to perform relatively similarly on simulations (29) and *in vitro* (30) studies. At least three possible explanations could explain these discrepancies: (1) the definition of the electrical onset of seizures can vary among epileptologists; (2) the changes in connectivity that culminate in seizures might begin before visually identified electrical onset, and (3) different frequency bands could act as distinct information transfer channels during seizures, which has been seldom investigated.

### Ictal vs. Interictal Networks

In addition to seizures, epilepsy is characterized by brief asymptomatic electrical discharges called interictal epileptiform discharges (IEDs). Given their sharp shape on electroencephalographic recordings and the large spatial overlap of their generators with the epileptic focus (31), IEDs might be an easier proxy than seizures to study the alterations of functional connectivity in epilepsy. Indeed, it was found that functional connectivity during IEDs might be informative for identifying the epileptic focus as they co-localize with the site of seizure onset (32–36). However, all of those studies evaluated functional connectivity in a relatively large window, which mixes the changes specific to IEDs with those preceding and following IEDs.

In the present study, we hypothesized that functional connectivity exhibits significant variations in the amplitude of synchronization time-locked to the appearance of IEDs, as recorded by intracranial macroelectrodes. In addition, we hypothesized that the epileptic focus displays smaller high-frequency synchrony during IEDs than other brain regions.

## Methods

### Patients

For this study, we retrospectively collected data from patients with pharmacoresistant epilepsy who were admitted at the epilepsy monitoring unit of the University of Montreal Hospital Center. Thirty-five consecutive patients undergoing iEEG recordings as part of their pre-surgical evaluation were recruited for this study. In case patients had more than one epilepsy surgery, only the first invasive investigation was considered in this study. The research project was approved by the University of Montreal hospital research center ethics committee (approval reference number: 18.016).

### Recordings

All iEEG recordings were acquired from a combination of subdural strip, grid (10 mm spacing, 2.3 mm diameter of exposed area per contact, AD-TECH, WI, USA) and depth electrodes (5 mm spacing, 1.6 mm contact per contact, AD-TECH, WI). iEEG signals were sampled at 2 kHz and digitized (eAMP 64 channel EEG amplifier, Stellate now Natus medical, CA). Electrode contacts were labeled according to timing of the epileptic activity they display during seizures into 3 classes: focus (F—involved at onset), propagation (P—involved after seizure spread), and silent (S—not involved). An expert epileptologist (D.K.N.) reviewed seizure traces and assigned class labels to each electrode contact.

### Data Processing

For each patient, we randomly selected a subset of IEDs among continuous recordings and extracted a three-second epoch around the positive peak of IEDs, as marked by an expert epileptologist (-2 to 1 s). To avoid sleep cycles' effect on IEDs, these were selected from awake state day recordings. An average of 51 IEDs were randomly selected per patient to provide a global picture of their interictal epileptiform activity. Data epochs were mirror band-pass filtered in consecutive frequency bands (theta-θ: 4-7Hz, alpha-α: 8-12Hz, beta-β: 12-30Hz, gamma1-γ1: 30-60Hz, and gamma2-γ2: 60-120Hz) with a finite impulse response (FIR) filter defined using a Kaiser window, as implemented in Brainstorm (37). The backward and forward filtering technique was used to keep the phase of signals intact. In order to avoid edge effects, only the [−1, 0.5] s window was considered for analysis. We then evaluated pairwise connectivity among iEEG electrode contacts within a frequency-adapted sliding window. We set the sliding window to two periods of the middle frequency per band (θ: 363 ms; α: 200 ms; β: 95 ms; γ1: 44 ms; γ2: 22 ms) and computed in each window the phase-locking value (PLV) (38), which is a measure of synchrony bounded between 0 (asynchrony) and 1 (synchrony). We thus obtained one N_e_ x N_e_ PLV-based synchrony matrix for each time window and frequency band, where N_e_ is the number of electrode contacts. For details regarding the mathematical basis of PLV, readers are referred to (38). In brief, PLV is a measure of synchrony which computes a degree of phase-locking between the components the components of 2 time series at each latency. The PLV at each time instant t is then computed as the average value:

$$\begin{array}{l} PLV_{t}=\frac{1}{N}\left|\overset{N}{\underset{n=1}{\sum }}exp\left(j\theta \left(t,n\right)\right)\right| \end{array}$$

where θ(*t, n*) is the difference between phases of the 2 given signals at time t and trial n, and N is the number of trials.

We recall that the size of the sliding window used for analysis of the time course of connectivity was frequency adaptive. Since phase locking of Hilbert-transformed signals assesses coupling among oscillators, the analysis window was selected in terms of cycles of oscillation. In this work, we chose 2 cycles of oscillation to balance the need for sufficient temporal resolution and signal-to-noise ratio.

### Dynamics of Global PLV-Based Synchrony

PLV-based synchrony values were averaged within each matrix to obtain a single time course for each IED and frequency band, representing the dynamics of global PLV-based synchrony. Since those matrices are symmetrical, only upper off-diagonal values were averaged. Time courses of PLV-based synchrony were then averaged over IEDs for each frequency band to reveal band-specific dynamics. In order to identify the time windows during which the global PLV-based synchrony level departs significantly from chance level, we designed a band-specific non-parametric statistical procedure. First, electrode signals were randomly shuffled across IEDs, such that electrode signal *X*_*i,k*_ from electrode *i* and IED *k* is replaced by the signal *X*_*i,l*_, where *l* is a IED index and *k*≠*l*. This procedure disrupts the connectivity structure among sensors while preserving the IED shape (39). Dynamics of global connectivity is then computed using the procedure described in the previous section. The shuffling operation is iterated 1,000 times to produce confidence intervals (99% percentile, corresponding to *p* < =0.01) on the global level of PLV-based synchrony at each time window.

To further ensure that observed dynamic changes of PLV-based synchrony are specific to IEDs, we repeated the described analysis on randomly selected epochs. For each patient, 50 markers were set randomly on the DC component of iEEG signals to avoid any bias toward epileptic activity. Epochs [-2, 1] s were then exported and analyzed.

### IED-Locked PLV-Based Synchrony Changes

**Figure 1:**
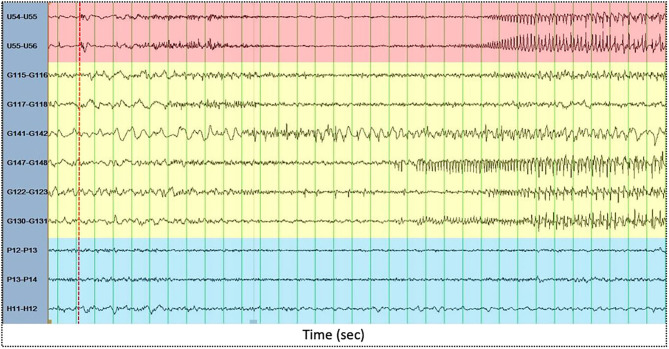
Illustrative examples of intracranial EEG ictal signals recorded by electrodes within the focus area (red), propagation area (yellow) and silent area (blue). The x-axis displays time (in seconds). Green vertical lines are spaced one second each. The red dashed vertical line displays electrical seizure onset.

**Figure 2:**
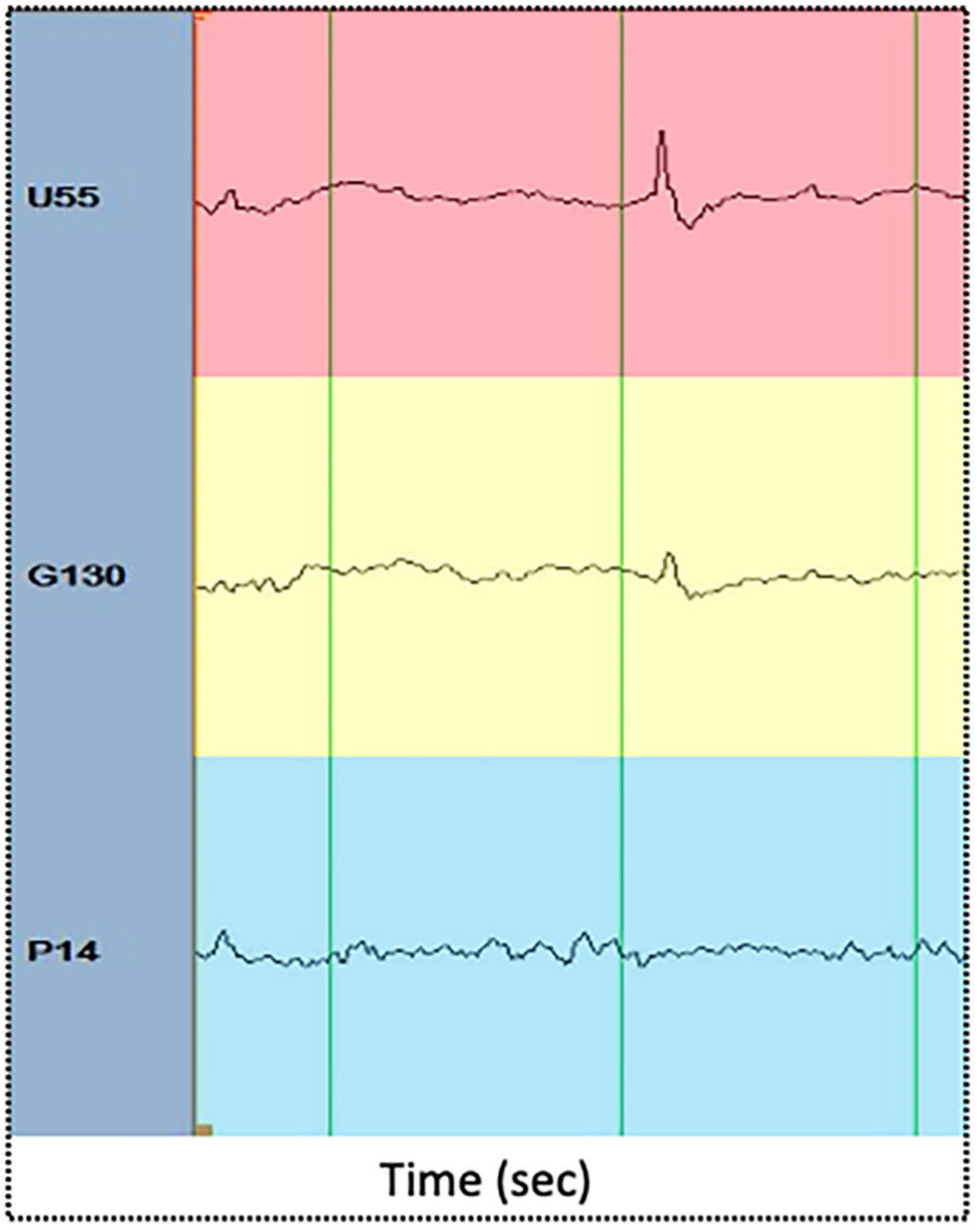
Illustrative examples of IEDs as recorded in the focus area (red), propagation area (yellow), and silent area (blue). The x axis displays time (in seconds). Green vertical lines are spaced one second each.

We then computed the dynamics of PLV-based synchrony according to the connectivity types: the links among electrodes in the focus (F-F), among electrodes in the propagation area (P-P), among electrodes in silent areas (S-S) and across areas (F-P, F-S, P-S) were averaged separately within each connectivity matrix. The focus area (or seizure onset zone), the propagation zone, and silent electrodes were determined by an expert epileptologist based on visual interpretation of seizure recordings. The focus area was defined as the one displaying the first unequivocal intracranial EEG sign of change from the background that leads to a clear sustained rhythmic discharge, without return to background activity. The propagation zone is composed of other electrodes (areas) showing ictal activity but with a delay with regard to the onset zone. Figure 1 shows illustrative examples of iEEG ictal signals recorded by electrodes within the focus area (red), propagation area (yellow) and silent area (blue) This electrode classification (based on ictal recordings) was then used to compute the dynamics of PLV-based synchrony during interictal epileptiform discharges. Figure 2 displays illustrative examples of IEDs as recorded in the focus area (red), propagation area (yellow), and silent area (blue).

The dynamics of PLV-based synchrony of each connectivity type were then averaged across IEDs to obtain an average time course. We then quantified the change in PLV-based synchrony at the IED peak relatively to a baseline window (-1 to−0.5s relative to IED marker) by computing a z-score ([IED peak—average of baseline] / std. of baseline). The PLV-based synchrony change scores involving the focus—F-F/F-P/F-S—and those not involving the focus—P-P/P-S/S-S—were analyzed separately.

We then assessed the relationship between the changes in PLV-based synchrony of each connectivity type and the surgical outcome of patients. We categorized patients as having either good (Engel I and II post-surgical outcome scores) or bad (Engel III or IV) and assessed the effect of surgical outcome and frequency bands on PLV-based synchrony changes. Since data patients with good and bad outcomes had unequal variances, we conducted non-parametric *t*-tests: for each frequency band, we computed the t-statistic to measure the difference of average PLV-based synchrony change between patients with good and bad outcomes. This statistic was compared against 100 000 surrogate t-statistic generated by randomly shuffling patients across groups. Differences between patients with respect to surgical outcome were considered significant if the t-statistic exceeded 500 random t-statistics, which corresponds to a Bonferroni-corrected 5% significance level.

## Results

### Patients

In total, 35 patients (15 female, age 34.0 ± 10.8 years—demographic and clinical data are provided in Table 1) were recruited and an average of 51 IEDs (average 51.5±8.0) were selected for each patient. Twenty-one and ten patients were categorized as having good and bad surgical outcomes, respectively. Follow-up was not available for four subjects and were thus excluded from the surgical outcome association analysis.

**Table 1:** Demographic and clinical characteristics of patients.

**ID**	**Age Range**	**Gender**	**Ep. Dur**.	**Total contacts (Depth)**	**Contacts localization**	**Resection**	**Engel**	**F.-U**.
1	[40-49]	M	19	84 (30)	Ins L, Temp L,	mTemp L	IA	4
2	[40-49]	M	4	128 (60)	mTemp L, mTemp R, Ins L, Ins R,Fr L, Fr R, Par L, Par R	mTemp L	IB	6
3	[40-49]	F	13	69 (15)	Ins R, mTemp R	Op-Ins R	IIIA	4
4	[50-59]	M	33	128 (8)	Fr R, Par R, Oc R, mTemp R, Fr L	Par R	IA	2.5
5	[40-49]	F	33	62 (10)	Op-Ins L, Temp L, Fr L	Op-Ins L	IA	1
6	[40-49]	M	24	114 (36)	mTemp L, Ins L, Temp L,	mTemp L	IIIA	3
7	[30-39]	F	21	48	Fr R, Fr L	Fr R	IID	2
8	[50-59]	M	43	134	Fr R, Fr L, Temp R,	Fr R	IA	5.5
9	[50-59]	M	50	108 (8)	Par L, Ins L, Temp L, Fr L, Fr R	Insula L	IB	7
10	[30-39]	F	32	118 (28)	Temp L, mTemp L,Ins R,Temp R, mTemp R	Insula R	IA	3.5
11	[50-59]	F	9	28 (20)	Ins L, Temp L	mTemp L	IA	4
12	[20-29]	M	17	130	Fr R, Fr L	Fr L	IA	6
13	[50-59]	M	16	106 (8)	mTemp L, Ins L, Fr L,	Fr L	IA	4
14	[70-79]	F	40	100 (4)	Temp R, ParR, Occ R, Op-Ins R, Fr R, Par R, mTemp R	mTemp R	IC	3
15	[30-39]	M	30	106 (12)	mTemp L, mTemp R, Ins R, Ins L, Par R, Par L, Fr L, Fr R, Temp R, Temp L,	Temp R	-	-
16	[30-39]	M	41	82 (16)	mTemp R, Ins R, Fr R, Temp R, Par R	Ins R	IV	0.3 ^**^
17	[30-39]	F	13	58	Occ L, Occ R, Temp R, Par R,	Occ R + Temp R + Par R	IA	2.5
18	[30-39]	M	23	118 (44)	Fr L, Temp L, OP-Ins L,	Fr R	IIIB	3
19	[20-29]	F	20	152 (8)	Par L, Occ L, Temp L, Fr L, Fr R	Occ L	IA	5
20	[20-29]	F	20	98 (5)	Fr R, Temp R, Ins R, mTemp R,	mTemp R	-	-
21	[40-49]	F	7	102 (24)	Op-Ins L, Temp L, Occ L, Par L, mTemp L.	Op-Ins L	IIIA	4
22	[20-29]	F	16	104	Fr R, Fr L	Fr L	IIIB	3
23	[50-59]	M	44	140	Fr R, Fr L, Par L, Temp L,	Fr L	IA	5
24	[30-39]	M	23	140	Fr R, Par R, Fr L	Premotor R	IV	4
25	[20-29]	F	8	92	Fr R, Fr L	Fr R	IA	4.5
26	[50-59]	M	34	108 (4)	Fr L, Par L	Fr L	IA	5.5
27	[30-39]	F	19	124 (8)	Fr R, Op-Ins R, Temp R,	Fr R + Ins R	IA	3
28	[20-29]	M	12	116 (8)	Ins L, Fr L, Temp L,	Fr L + Temp L	-	-
29	[30-39]	M	5	112 (8)	Fr L, Ins L, Temp L,	Fr + Ins R	IIIA	2
30	[40-49]	M	35	100 (12)	Fr R, Temp R, Par R, Ins R	Fr-Op R + Ins R	IA	3
31	[30-39]	M	26	126 (32)	Ins L, Fr L, Temp L, Par L, mTemp L	-	-	-
32	[20-29]	F	12	74	Fr L, Par L, Temp L	inf-Rol L	IA	2
33	[50-59]	F	47	106 (14)	Ins L, Op-Ins L, Temp L, Par, Fr L	mTemp L	IIIA	2
34	[50-59]	M	31	96 (28)	Ins L, OP-Ins L, Temp L, Par L, mTemp L	Op-Ins L	IA	2
35	[20-29]	M	20	108 (16)	Fr R, Temp R, Par R, Occ R, Ins R	Fr R	IVA	2

*Reported age corresponds to age at surgery (mTemp: mesio-temporal; Op-Ins: operculo-insular; biTemp: bitemporal; Fr: frontal; Ins: insular; Occ: occipital; Par: Parietal R: right; L: left; Fr-Op: frontal operculum; inf-Rol: inferior rolandic*;

** *: case of SUDEP; F.-U.: follow-up in number of years)*.

### Dynamics of Global PLV-Based Synchrony

**Figure 3:**
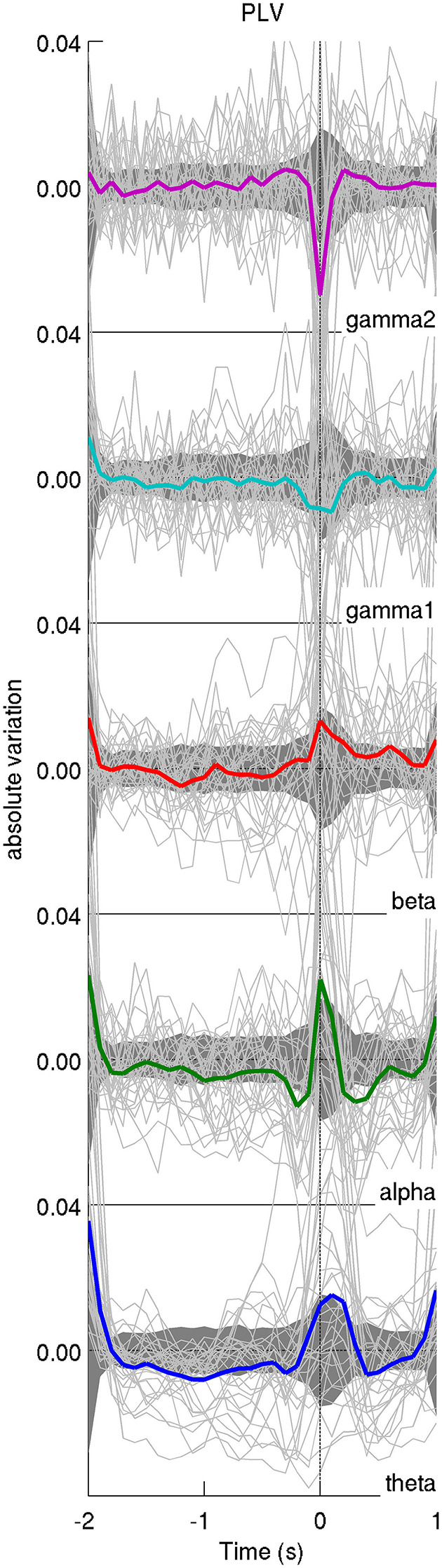
Average time course of the iEEG functional connectivity. The average PLV across pairs of iEEG contacts for each patient is represented with a light solid line and the average across patients is displayed in thick colored lines. Confidence intervals for the average across patients are displayed as a dark shaded area. The time axis is centered on the peak of IEDs (time 0). In general, the average functional connectivity, as measured with the phase-locking value (PLV), increases at IED peak for lower frequency bands (<30 Hz) and decreases for higher frequency bands (>30 Hz). The y-axis displays absolute variation in terms of z-score.

**Figure 4:**
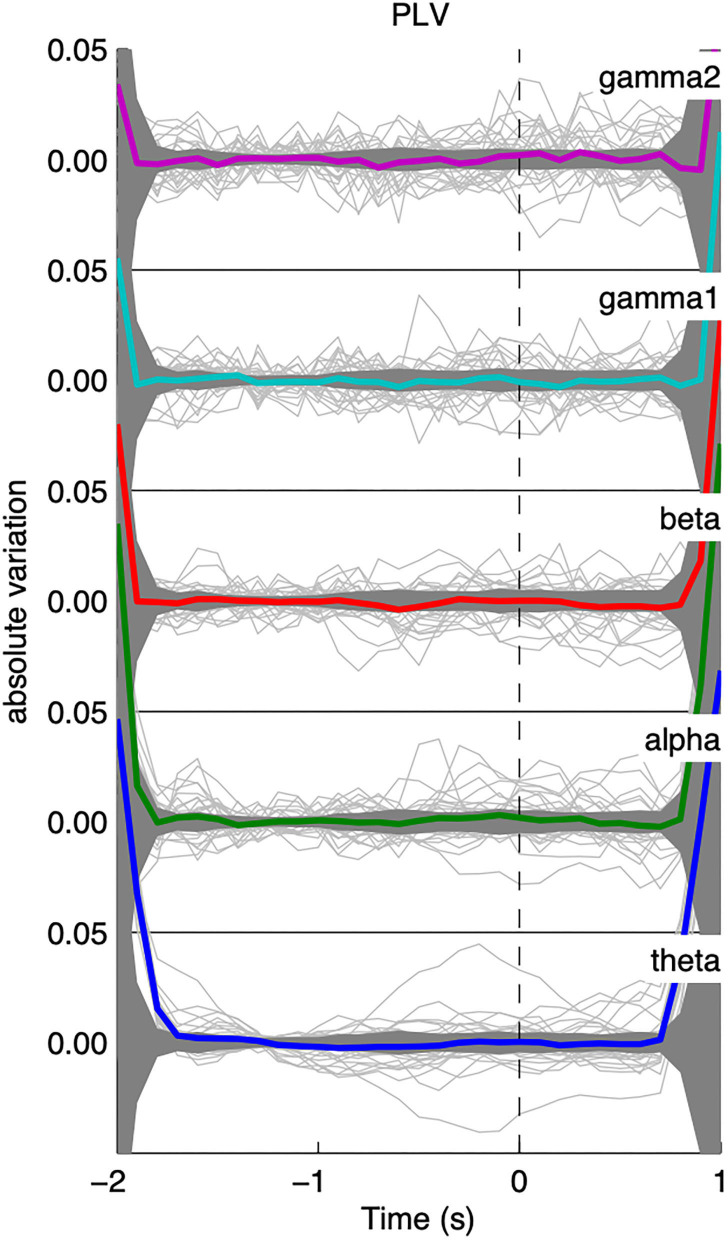
Average time course of the shuffled iEEG functional connectivity; results were only flat noise and no change in connectivity was observed for all frequency bands.

As can be seen from Figure 3, the global level of PLV-based synchrony changes in a frequency-dependent manner. Low frequency bands (θ, α, β) display an increase in PLV-based synchrony time-locked to the appearance of the IEDs while high frequency bands display a decrease in PLV-based synchrony. However, those changes exceed the confidence interval only for the γ2 band. PLV-based synchrony changes for low-frequency bands were not statistically significant. Interestingly, such changes were absent from global PLV-based synchrony during randomly selected epochs, for any frequency band (Figure 4).

We found no significant interplay between low and high frequency bands. By correlating the average relative changes in functional connectivity across low and high frequency bands (theta/alpha/beta X gamma1/gamma2) across patients, we found no statistically significant linear associations.

### IED Changes of PLV-Based Synchrony According to Connectivity Type

**Figure 5:**
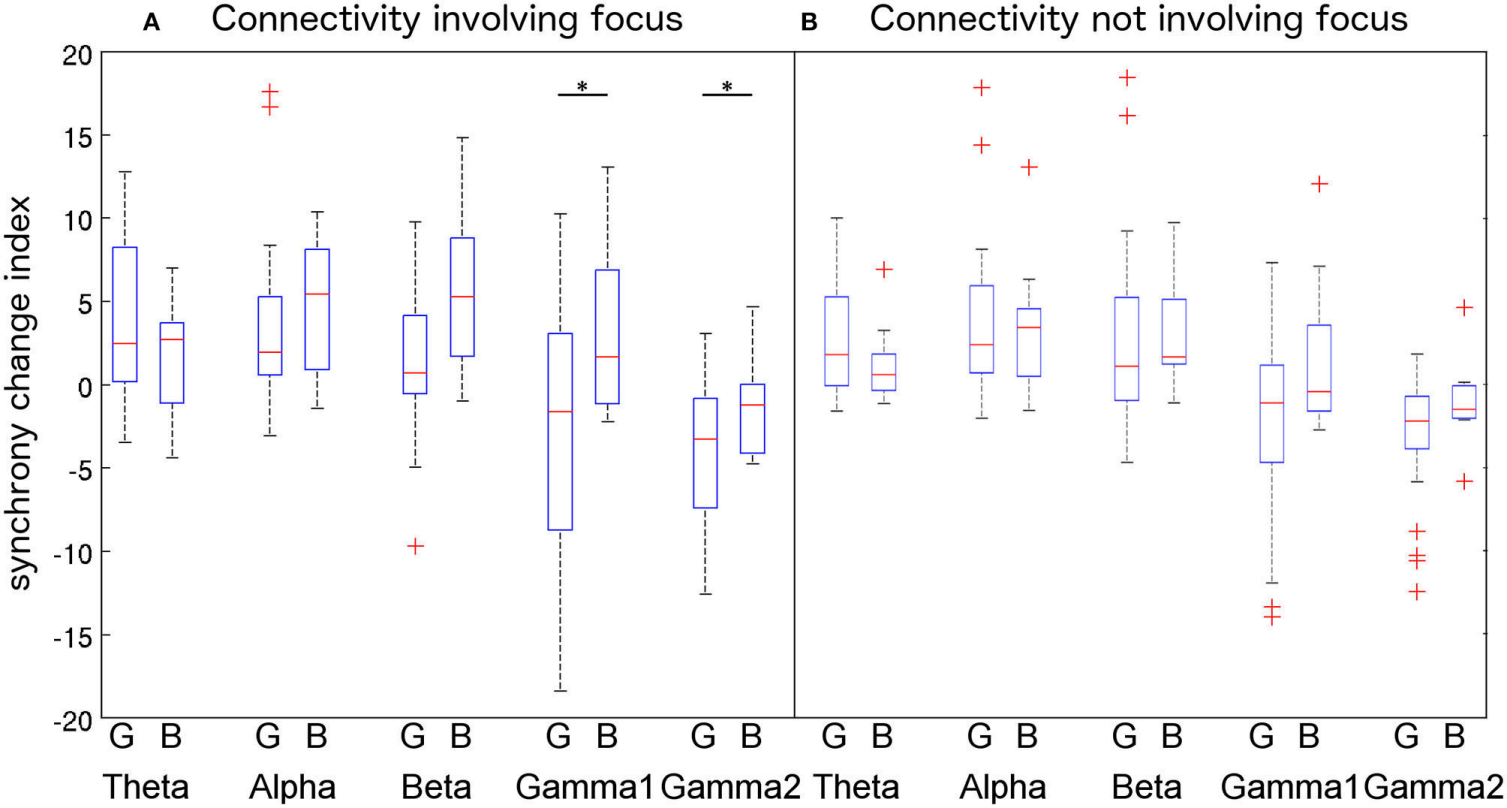
Comparison of PLV variations among the (good outcome—G vs. bad outcome—B) patients groups for each frequency band using a 2-way ANOVA. Connectivity involving the epileptic focus (F-F, F-P, and F-S connections, **A**) increases in lower frequency bands and decrease in higher frequency bands. Differences between patients groups reach significance at the 5% level for gamma1 and gamma2 bands. Connectivity not involving the focus (P-P, P-S, and S-S, **B**) shows similar trends but differences were not significant. Outliers are represented with a “+” symbol. Significant differences are represented with a “*” symbol.

We compared changes of PLV-based synchrony at the IED peak relative to a preceding baseline period separately for connectivity involving the focus and connectivity not involving the focus:

When analyzing PLV-based synchrony involving the focus (Figure 5, left), non-parametric pairwise *t*-tests revealed that IED-locked high-frequency (γ1: *p* = 0.0088; γ2: *p* = 0.0183) PLV-based synchrony is significantly different between patients with good and bad outcomes. However, those differences did not survive Bonferroni correction (γ1: *p* = 0.088; γ2: *p* = 0.18). No significant differences in low-frequency IED-locked PLV-based synchrony were found between patients with good and bad outcomes (θ: *p* = 0.95, α: *p* = 0.26, β: *p* = 0.31). We then averaged IED-locked PLV-based synchrony changes within low (θ+α+β) and high (γ1+γ2) frequencies and repeated the non-parametric test. We found that IED-locked high-frequency PLV-based synchrony was significantly lower in patients with good than in patients with bad outcome (*p* = 0.023, corrected) while no difference was found for low-frequency PLV-based synchrony (*p* = 0.59, uncorrected).

When analyzing PLV-based synchrony not involving the focus (Figure 5, right), we found that neither high (γ1: *p* = 0.43; γ2: *p* = 0.48—corrected) nor low (θ: *p* = 0.93, α: *p* = 0.67, β: *p* = 0.46—uncorrected) frequency IED-locked PLV-based synchrony differed between patients with good and bad outcomes. After averaging PLV-based synchrony changes within bands, differences between patients with good and bad outcomes were not significant (low freq.: *p* = 0.92; high freq.: *p* = 0.092—corrected).

### Illustrative Cases

**Figure 6:**
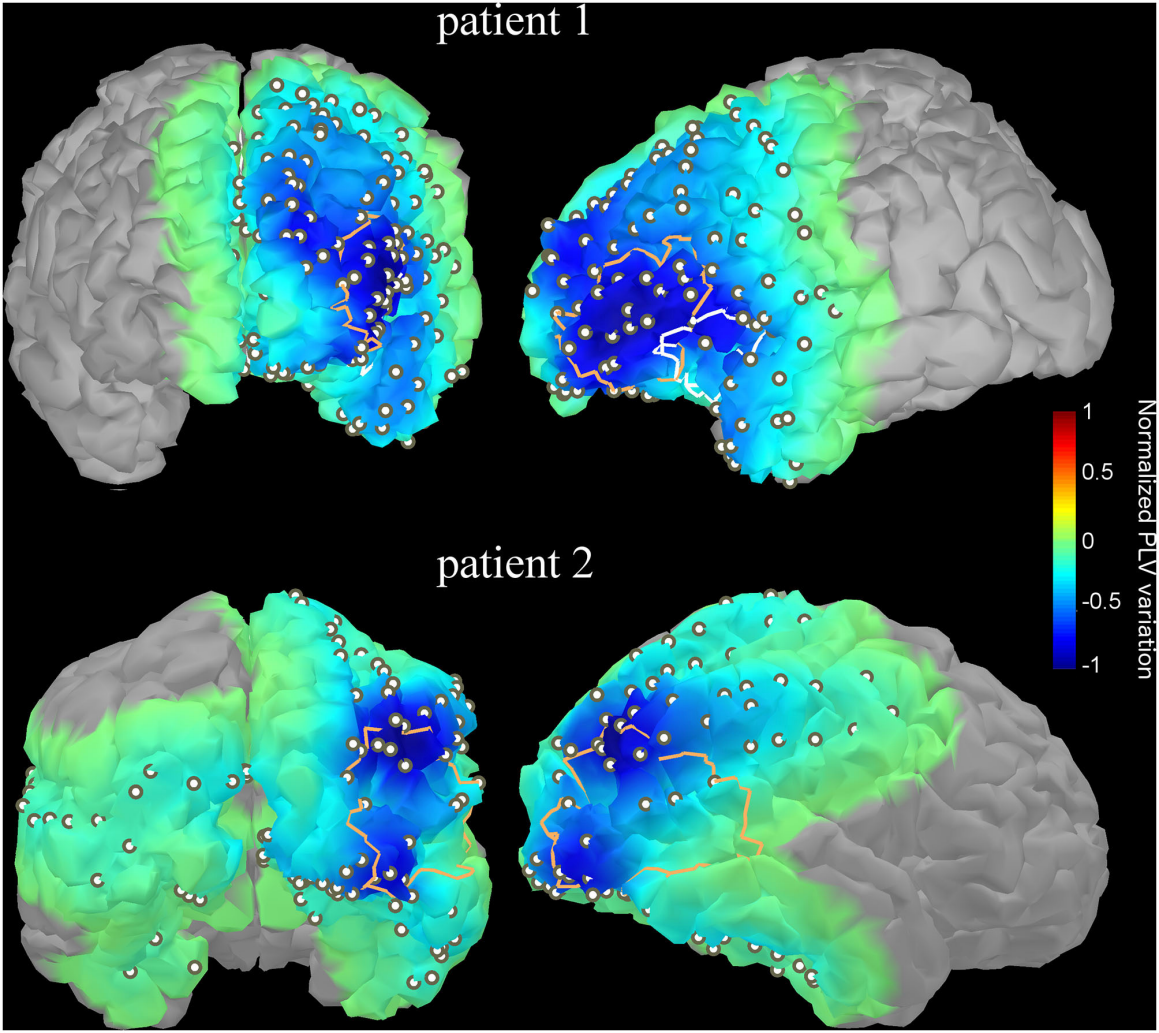
Variation of functional connectivity in the high gamma band (60–120 Hz) as measured with PLV index at the IED peak with respect to baseline (-1 to−0.5 s) for two illustrative patients. The variation index was computed for each electrode contact then interpolated over the underlying cortical surface using an in-house algorithm. The resection site targeted by a later surgery is overlaid as a thick contour. The first patient (upper row) had 2 surgeries; the first one targeted mainly the anterior insula (white contour line) while the maximal decrease of PLV was found in the lateral inferior frontal gyrus. The second surgery (orange contour line) targeted left orbitofrontal and frontal lateral regions, which was concordant with the sites of maximal PLV variation. For the second patient, the resected site (orange contour line) overlaps with the two sites with maximal PLV variation. The PLV projection onto the modeled cortex was threshold; only points on the projection of the cortex which account for 95% of the variance were kept.

*Patient 1* is a 35 year-old male with persistent nocturnal seizures (2–3 per day) despite eight antiepileptic drug trials. A presurgical evaluation revealed on surface EEG left fronto-temporal IEDs while ictal single-photon emission computed tomography (SPECT) and interictal positron emission tomography (PET) imaging showed, respectively hyperperfusion and hypometabolism over the left inferior frontal gyrus and anterior insula. MEG dipole imaging of IEDs showed a dense cluster of dipoles over the left superior temporal gyrus, anterior insula and left inferior frontal gyrus. IEEG recordings showed frequent interictal spiking activity in the anterior insula and broad onset of seizures, including the insula, the inferior frontal gyrus, the orbito-frontal gyrus and the superior temporal gyrus. The patient subsequently underwent an insulectomy (see Figure 6, top) that rendered him seizure-free for 6 months prior to recurrence. Interestingly, the resection site does not overlap with the site of maximal decrease of functional connectivity in the gamma2 band at the peak of IEDs (Figure 6, top), which was rather located in the lateral inferior frontal gyrus. Importantly, a second surgery targeting the lateral inferior frontal gyrus and orbitofrontal cortex subsequently rendered the patient seizure-free (follow-up of 2 years).

*Patient 2* is a 23-year-old female with pharmacoresistant non-lesional right occipital lobe epilepsy. Scalp EEG revealed frequent spiking over bilateral temporo-occipital regions with right predominance. Seizures started with low-voltage fast activity over the right occipito-temporal regions. Interictal PET and ictal SPECT imaging revealed, respectively hypometabolism and hyperperfusion over the same regions. MEG dipole imaging revealed a dense cluster over the right occipito-temporal region. Those observations were confirmed with iEEG recordings that showed frequent spiking over occipital and posterior temporal contacts. Surgical resection of the occipito-temporal junction (Figure 6, bottom), which overlapped with two “hot spots” showing maximal decrease of functional connectivity at IED peak, rendered the patient seizure-free (follow-up of 2 years).

## Discussion

In this work, we investigated the role of PLV-based synchrony during the generation of IEDs at the macroscopic scale of brain networks using a relatively large cohort of patients (*N* = 35) with an average of 51 IEDs per patient. In addition, we explored the link between changes in levels of PLV-based synchrony at the IED peak and the surgical outcome of patients.

### Network Desynchronization in Epilepsy

We found that the high frequency (γ1 + γ2) component of iEEG signals during interictal epileptiform discharges is associated with a statistically significant transient decrease in functional connectivity, time locked to the peak of the IEDs. Our results are in line with observations made during seizures; Mormann et al. found that seizures are consistently preceded by a state of hyposynchronization involving both the epileptic focus and distant brain areas (24). Burns et al. demonstrated a specific association between decreased synchronization at seizure onset and the epileptic focus, as surgical resection of the regions that display desynchronization at seizure onset correlated with good outcome in patients with refractory epilepsy (40). Our results are concordant with that study, as we showed significant relationship between the degree of desynchronization of the resected region with the surgical outcome of patients. Taken together with previous studies on long-range synchronization in epilepsy, our results suggest that transient desynchronization is observed during interictal epileptiform discharges.

Importantly, our study showed that decreases in PLV-based synchrony during IEDs were restricted to the gamma 1 and gamma 2 frequency bands. By recording simultaneously unit activity in pairs of neurons and the local field potential in slice preparations of rats hippocampus, Netoff and Schiff observed that seizure initiation is marked by a decrease of synchrony both between pairs of neurons and between individual neurons and the local field potential (30). This transient decrease in synchrony was specific to seizure initiation since epileptic bursts were associated with increased synchrony both between individual neurons and between neurons and the local field potential. In humans, simultaneous recordings of multiunit activity using microelectrode arrays and local field potentials revealed that microscopic seizures are initiated in very focal regions while the majority of the clinically defined seizure onset zone is silent. Transition to macroscopic seizures is thought to result from the coalescence of small seizing territories into a synchronized neural mass. At this stage, synchrony between the seizing cortex and the silent regions of the brain drops below baseline levels (41). Our results and those from studies showing transient desynchronization (40, 42, 43) at seizure onset are in line with this explanation.

The emergence of a hypersynchronous state, i.e., seizures, in weakly coupled networks has also been studied using numerical simulations. Nishikawa and Motter showed that any network under quantized total interactions could reach hypersynchronous states (44). In their simulations, they showed that weakly coupled networks can reach hypersynchrony after a phase of relative disconnection during which some specific links are removed (44), which supports the hypothesis that functional decoupling is a possible mechanism for seizure initiation.

### Functional Isolation Through Desynchronization

More than fifty years ago, Cannon proposed the “Law of denervation” to account for increased neuronal sensitivity following deprivation of the nervous connections from which they routinely receive inputs (45). In a series of observations on neurons in the spinal cord (46) and the cortex (47, 48) it was demonstrated that isolation of a patch of neurons after incision of its lateral connections increases its sensitivity to both chemical agents and electrical stimulation, thereby lowering the threshold for eliciting epileptiform activity. Can desynchronization transiently mimic functional isolation in human cortex such as to promote epileptiform activity?

Warren et al. used iEEG to compare the resting-state functional connectivity of the epileptic focus in epileptic patients to that of homologous regions in control patients suffering from facial pain injury and implanted with depth electrodes (43). They found that connectivity of the epileptic focus with surrounding regions was significantly reduced as compared to both intra-focus connections and homologous connections in control patients (43). They concluded that the epileptic focus was “functionally disconnected” from the rest of the brain in epilepsy. Recently, Burns et al. supported this view by analyzing seizures in human patients. They found that seizures can be robustly described with a finite set of states including an “isolated focus” state during which the focus exhibits decreased connectivity with the rest of the brain (40). Interestingly, patients who display the isolated focus state in early stages of seizures are those with good postsurgical outcomes while patients who do not display such a state have bad outcomes.

In line with that study, we found in our work that IED-locked high frequency (γ1+γ2) PLV-based synchrony was significantly lower in patients with good outcome than in patients with bad outcome. In other words, patients with good postsurgical outcome have a significantly more disconnected focus during IEDs than patients with bad outcome. We speculate that the epileptic focus is under the influence of resting-state networks during interictal periods and transient desynchronization is necessary to achieve transition toward the spiking and ictal states. More specifically, we found that IED-locked gamma-band PLV-based synchrony was significantly lower in patients with good outcome than in patients with bad outcome when analyzing connectivity within the focus (*p* < 0.05, corrected). We were not able to reveal differences in discriminative power (among patients with good vs. bad outcomes) between γ1 and γ2 frequency bands.

Clearly, we must remain prudent before translating this work into clinical practice since findings are based on group analyses using retrospective data from a modest number of patients. Further studies with larger cohorts of patients are required to better characterize the contribution of gamma (and subdivisions of gamma) frequency bands. Furthermore, in this work, electrodes were labeled according to the timing of the epileptic activity they displayed during seizures as classified by an expert epileptologist. Exploring how changing electrode class can impact the observed results could be an interesting future avenue. Given the sample size of our study, we could not investigate the effect of antiseizure medications on functional connectivity patterns or postoperative seizure control.

### Frequency-Specific Networks in Epilepsy

Our findings suggest band-specific changes in the time course of functional connectivity during IEDs. Although changes were only significant for the γ2 band, a preliminary distinction between frequency bands can be observed by comparing higher (>30 Hz) and lower (<30 Hz) bands, which respectively displayed decrease and increase in connectivity at the IED peak. This distinction is expected since they have distinct neural correlates and physiological roles in the healthy brain. Indeed, the low frequency range of the local field potentials reflects mainly synaptic activity and was associated with memory (theta band) (49, 50), arousal (alpha band) (51) and motor processing (beta band) (52), while higher frequencies reflect partly neural spiking activity (53) and were associated with higher cognitive functions such as visual (54) and auditory (55) perception. In epilepsy, the spectral power of high frequencies (>50 Hz) was shown to increase prior to seizure initiation in both humans (56–58) and animal models (59).

Relative contributions of synchronized neural activity within specific frequency bands to epileptiform activity are yet poorly understood. Most of the previous studies investigating epilepsy-related changes in functional connectivity using EEG (either intracranial or scalp) assessed coupling in the broad band signal, which is mainly dominated by lower frequencies given the well-described 1/f shape of the power density spectrum on electrophysiological signals. In addition, we found a distinction between high (>60 Hz) and low (<60 Hz) gamma bands to connectivity changes. This distinction is in line with changes in the power spectrum density of EEG prior to seizures, which shows a bimodal distribution with two statistically independent peaks in the (low: 30–60 Hz, high: 60–120 Hz) gamma range (57).

## Conclusion

This study suggests that the emergence of interictal epileptiform activity is time-locked with a decrease in functional connectivity in the γ2 frequency range. Moreover, IED-locked gamma band PLV-based synchrony was significantly lower in patients with good than in patients with bad outcome recalling to a network configuration in which the epileptogenic focus is functionally isolated from the rest of the brain. While further studies with a larger cohort of patients are required, our results show promise for the design of quantitative methods capable of quantitatively localizing the epileptic focus.

## Data Availability Statement

The datasets generated for this study will not be made publicly available as this is not allowed by the Ethical Research Committee. Requests to access the data should be sent to the corresponding author.

## Ethics Statement

The studies involving human participants were reviewed and approved by University of Montreal Hospital Research Center Ethical Committee. Written informed consent for participation was not required for this study in accordance with the national legislation and the institutional requirements.

## Author Contributions

EB and YZ wrote the main manuscript text. EB formatted the manuscript and submitted it. YZ and MR retrieved the data. YZ performed the analysis. FL and PP acted as consultants for the study design, signal processing, and statistical analysis. DKN annotated the interictal epileptiform discharges and was the principal investigator of this study. All authors reviewed the manuscript. All authors contributed to the article and approved the submitted version.

## Conflict of Interest

The authors declare that the research was conducted in the absence of any commercial or financial relationships that could be construed as a potential conflict of interest.

## References

[1] Desmond JE Sum JM Wagner AD Demb JB Shear PK Glover GH Functional MRI measurement of language lateralization in Wada-tested patients. Brain. (1995) 118:1411–9. 10.1093/brain/118.6.1411 8595473

[2] Geschwind N. Anatomical and functional specialization of the cerebral hemispheres in the human. Bull Mem Acad R Med Belg. (1979)134:286–97. 534784

[3] Roelfsema PR Engel AK Konig P Singer W. The role of neuronal synchronization in response selection: a biologically plausible theory of structured representations in the visual cortex. J Cogn Neurosci. (1996) 8:603–25. 10.1162/jocn.1996.8.6.603 23961987

[4] Varela F Lachaux JP Rodriguez E Martinerie J. The brainweb: phase synchronization and large-scale integration. Nat Rev Neurosci. (2001) 2:229–39. 10.1038/35067550 11283746

[5] He BJ Shulman GL Snyder AZ Corbetta M. The role of impaired neuronal communication in neurological disorders. Curr Opin Neurol. (2007) 20:655–60. 10.1097/WCO.0b013e3282f1c720 17992085

[6] Spencer SS. Neural networks in human epilepsy: evidence of and implications for treatment. Epilepsia. (2002) 43:219–27. 10.1046/j.1528-1157.2002.26901.x 11906505

[7] Clemens B Puskas S Besenyei M Spisak T Opposits G Hollody K. Neurophysiology of juvenile myoclonic epilepsy: EEG-based network and graph analysis of the interictal and immediate preictal states. Epilepsy Res. (2013) 106:357–69. 10.1016/j.eplepsyres.2013.06.017 23886656

[8] Elshahabi A Klamer S Sahib AK Lerche H Braun C Focke NK. Magnetoencephalography reveals a widespread increase in network connectivity in idiopathic/genetic generalized epilepsy. PLoS ONE. (2015) 10:e0138119. 10.1371/journal.pone.0138119 26368933 PMC4569354

[9] Maccotta L He BJ Snyder AZ Eisenman LN Benzinger TL Ances BM. Impaired and facilitated functional networks in temporal lobe epilepsy. Neuroimage Clin. (2013) 2:862–72. 10.1016/j.nicl.2013.06.011 24073391 PMC3777845

[10] Morgan VL Rogers BP Sonmezturk HH Gore JC Abou-Khalil B. Cross hippocampal influence in mesial temporal lobe epilepsy measured with high temporal resolution functional magnetic resonance imaging. Epilepsia. (2011) 52:1741–9. 10.1111/j.1528-1167.2011.03196.x 21801166 PMC4428312

[11] Antony AR Alexopoulos AV González-Martínez JA Mosher JC Jehi L Burgess RC. Functional connectivity estimated from intracranial EEG predicts surgical outcome in intractable temporal lobe epilepsy. PLoS ONE. (2013) 8:e77916. 10.1371/journal.pone.0077916 24205027 PMC3813548

[12] Bettus G Bartolomei F Confort-Gouny S Guedj E Chauvel P Cozzone PJ. Role of resting state functional connectivity MRI in presurgical investigation of mesial temporal lobe epilepsy. J Neurol Neurosurg Psychiatry. (2010) 81:1147–54. 10.1136/jnnp.2009.191460 20547611

[13] Bettus G Wendling F Guye M Valton L Regis J Chauvel P. Enhanced EEG functional connectivity in mesial temporal lobe epilepsy. Epilepsy Res. (2008) 81:58–68. 10.1016/j.eplepsyres.2008.04.020 18547787

[14] Pittau F Grova C Moeller F Dubeau F Gotman J. Patterns of altered functional connectivity in mesial temporal lobe epilepsy. Epilepsia. (2012) 53:1013–23. 10.1111/j.1528-1167.2012.03464.x 22578020 PMC3767602

[15] Schevon CA Cappell J Emerson R Isler J Grieve P Goodman R. Cortical abnormalities in epilepsy revealed by local EEG synchrony. Neuroimage. (2007) 35:140–8. 10.1016/j.neuroimage.2006.11.009 17224281 PMC1994936

[16] Dai Y Zhang W Dickens DL He B. Source connectivity analysis from MEG and its application to epilepsy source localization. Brain Topogr. (2012) 25:157–66. 10.1007/s10548-011-0211-0 22102157 PMC3299922

[17] Bou Assi E Rihana S Nguyen DK Sawan M. Effective connectivity analysis of iEEG and accurate localization of the epileptogenic focus at the onset of operculo-insular seizures. Epilepsy Res. (2019) 152:42–51. 10.1016/j.eplepsyres.2019.02.006 30878795

[18] Elshoff L Muthuraman M Anwar AR Deuschl G Stephani U Raethjen J. Dynamic imaging of coherent sources reveals different network connectivity underlying the generation and perpetuation of epileptic seizures. PLoS ONE. (2013) 8:e78422. 10.1371/journal.pone.0078422 24194931 PMC3806832

[19] van Mierlo P Carrette E Hallez H Raedt R Meurs A Vandenberghe S. Ictal-onset localization through connectivity analysis of intracranial EEG signals in patients with refractory epilepsy. Epilepsia. (2013) 54:1409–18. 10.1111/epi.12206 23647147

[20] Amor F Baillet S Navarro V Adam C Martinerie J Quyen Mle. V. Cortical local and long-range synchronization interplay in human absence seizure initiation. Neuroimage. (2009) 45:950–62. 10.1016/j.neuroimage.2008.12.011 19150654

[21] Parra J Kalitzin SN Iriarte J Blanes W Velis DN Lopes da Silva FH. Gamma-band phase clustering and photosensitivity: is there an underlying mechanism common to photosensitive epilepsy and visual perception? Brain. (2003) 126:1164–72. 10.1093/brain/awg109 12690055

[22] Bartolomei F Wendling F Bellanger JJ Regis J Chauvel P. Neural networks involving the medial temporal structures in temporal lobe epilepsy. Clin Neurophysiol. (2001) 112:1746–60. 10.1016/S1388-2457(01)00591-0 11514258

[23] Kramer MA Eden UT Kolaczyk ED Zepeda R Eskandar EN Cash SS. Coalescence and fragmentation of cortical networks during focal seizures. J Neurosci. (2010) 30:10076–85. 10.1523/JNEUROSCI.6309-09.2010 20668192 PMC2927849

[24] Mormann F Andrzejak RG Kreuz T Rieke C David P Elger CE. Automated detection of a preseizure state based on a decrease in synchronization in intracranial electroencephalogram recordings from epilepsy patients. Phys Rev E Stat Nonlin Soft Matter Phys. (2003) 67:021912. 10.1103/PhysRevE.67.021912 12636720

[25] Mormann F Lehnertz K David PE Elger C. Mean phase coherence as a measure for phase synchronization and its application to the EEG of epilepsy patients. Physica D. (2000) 144:358–69. 10.1016/S0167-2789(00)00087-712636720

[26] Schindler K Elger CE Lehnertz K. Increasing synchronization may promote seizure termination: evidence from status epilepticus. Clin Neurophysiol. (2007) 118:1955–68. 10.1016/j.clinph.2007.06.006 17644031

[27] Schindler KA Bialonski S Horstmann MT Elger CE Lehnertz K. Evolving functional network properties and synchronizability during human epileptic seizures. Chaos. (2008) 18:33119. 10.1063/1.2966112 19045457

[28] Varotto G Tassi L Franceschetti S Spreafico R Panzica F. Epileptogenic networks of type II focal cortical dysplasia: a stereo-EEG study. Neuroimage. (2012) 61:591–8. 10.1016/j.neuroimage.2012.03.090 22510255

[29] Wendling F Ansari-Asl K Bartolomei F Senhadji L. From EEG signals to brain connectivity: a model-based evaluation of interdependence measures. J Neurosci Methods. (2009) 183:9–18. 10.1016/j.jneumeth.2009.04.021 19422854

[30] Netoff TI Schiff SJ. Decreased neuronal synchronization during experimental seizures. J Neurosci. (2002) 22:7297–307. 10.1523/JNEUROSCI.22-16-07297.2002 12177225 PMC6757884

[31] Hufnagel A Dumpelmann M Zentner J Schijns O Elger CE. Clinical relevance of quantified intracranial interictal spike activity in presurgical evaluation of epilepsy. Epilepsia. (2000) 41:467–78. 10.1111/j.1528-1157.2000.tb00191.x 10756415

[32] Englot DJ Rolston JD Wang DD Kirsch HE Nagarajan SS Chang EF 206 spikes, slowing, and functional connectivity: multimodal magnetoencephalography in epilepsy surgery. Neurosurgery. (2016) 63:181 10.1227/01.neu.0000489775.61051.9c

[33] Iannotti GR Grouiller F Centeno M Carmichael DW Abela E Wiest R. Epileptic networks are strongly connected with and without the effects of interictal discharges. Epilepsia. (2016) 57:1086–96. 10.1111/epi.13400 27153929

[34] Jmail N Gavaret M Bartolomei F Chauvel P Badier JM Benar CG. Comparison of brain networks during interictal oscillations and spikes on magnetoencephalography and intracerebral EEG. Brain Topogr. (2016) 29:752–65. 10.1007/s10548-016-0501-7 27334988

[35] Luo C An D Yao D Gotman J. Patient-specific connectivity pattern of epileptic network in frontal lobe epilepsy. NeuroImage. (2014) 4:668–75. 10.1016/j.nicl.2014.04.006 24936418 PMC4053646

[36] Malinowska U Badier JM Gavaret M Bartolomei F Chauvel P Benar CG. Interictal networks in magnetoencephalography. Hum Brain Mapp. (2014) 35:2789–805. 10.1002/hbm.22367 24105895 PMC6869550

[37] Tadel F Baillet S Mosher JC Pantazis D Leahy RM. Brainstorm: a user-friendly application for MEG/EEG analysis. Comp Intell Neurosci. (2011) 2011:13. 10.1155/2011/879716 21584256 PMC3090754

[38] Lachaux JP Rodriguez E Martinerie J Varela FJ. Measuring phase synchrony in brain signals. Hum Brain Mapp. (1999) 8:194–208. 10.1002/(SICI)1097-0193(1999)8:4<194::AID-HBM4>3.0.CO;2-C 10619414 PMC6873296

[39] Zerouali Y Pouliot P Robert M Mohamed I Bouthillier A Lesage F. Magnetoencephalographic signatures of insular epileptic spikes based on functional connectivity. Hum Brain Mapp. (2016) 37:3250–61. 10.1002/hbm.23238 27220112 PMC6867448

[40] Burns SP Santaniello S Yaffe RB Jouny CC Crone NE Bergey GK. Network dynamics of the brain and influence of the epileptic seizure onset zone. Proc Natl Acad Sci. (2014) 111:E5321. 10.1073/pnas.1401752111 25404339 PMC4267355

[41] Schevon CA Weiss SA McKhann G. Jr. Goodman RR Yuste R. Evidence of an inhibitory restraint of seizure activity in humans. Nat Commun. (2012) 3:1060. 10.1038/ncomms2056 22968706 PMC3658011

[42] Khambhati AN Davis KA Oommen BS Chen SH Lucas TH Litt B. Dynamic network drivers of seizure generation, propagation and termination in human neocortical epilepsy. PLOS Comp Biol. (2015) 11:e1004608. 10.1371/journal.pcbi.1004608 26680762 PMC4682976

[43] Warren CP Hu S Stead M Brinkmann BH Bower MR Worrell GA. Synchrony in normal and focal epileptic brain: the seizure onset zone is functionally disconnected. J Neurophysiol. (2010) 104:3530–9. 10.1152/jn.00368.2010 20926610 PMC3007634

[44] Nishikawa T Motter AE. Network synchronization landscape reveals compensatory structures, quantization, and the positive effect of negative interactions. Proceed Natl Acad Sci USA. (2010) 107:10342. 10.1073/pnas.0912444107 20489183 PMC2890837

[45] Cannon WB A law of denervation. Am J Med Sci. (1939) 1:198 10.1097/00000441-193912000-00001

[46] Cannon WB Haimovici H The sensitization of motoneurones by partial “Denervation”. Am J Physiol. (1939) 126:731–40. 10.1152/ajplegacy.1939.126.3.731

[47] Echlin FA. The supersensitivity of chronically “isolated” cerebral cortex as a mechanism in focal epilepsy. Electroencephalogr Clin Neurophysiol. (1959) 11:697–722. 10.1016/0013-4694(59)90110-5 13819191

[48] Echlin FA Battista A. Epileptiform seizures from chronic isolated cortex. Arch Neurol. (1963) 9:154–70. 10.1001/archneur.1963.00460080064009 14048164

[49] Axmacher N Henseler MM Jensen O Weinreich I Elger CE Fell J. Cross-frequency coupling supports multi-item working memory in the human hippocampus. Proc Natl Acad Sci USA. (2010) 107:3228. 10.1073/pnas.0911531107 20133762 PMC2840289

[50] Kaplan R Bush D Bonnefond M Bandettini PA Barnes GR Doeller CF. Medial prefrontal theta phase coupling during spatial memory retrieval. Hippocampus. (2014) 24:656–65. 10.1002/hipo.22255 24497013 PMC4028411

[51] Bollimunta A Mo J Schroeder CE Ding M. Neuronal mechanisms and attentional modulation of corticothalamic alpha oscillations. J Neurosci. (2011) 31:4935–43. 10.1523/JNEUROSCI.5580-10.2011 21451032 PMC3505610

[52] Khanna P Carmena JM. Neural oscillations: beta band activity across motor networks. Curr Opin Neurobiol. (2015) 32:60–7. 10.1016/j.conb.2014.11.010 25528615

[53] Buzsáki G Anastassiou CA Koch C. The origin of extracellular fields and currents - EEG. ECoG, LFP and spikes. Nat Rev Neurosci. (2012) 13:407. 10.1038/nrn3241 22595786 PMC4907333

[54] Rodriguez E George N Lachaux JP Martinerie J Renault B Varela FJ. Perception's shadow: long-distance synchronization of human brain activity. Nature. (1999) 397:430–33. 10.1038/17120 9989408

[55] Steinmann S Leicht G Ertl M Andreou C Polomac N Westerhausen R. Conscious auditory perception related to long-range synchrony of gamma oscillations. Neuroimage. (2014) 100:435–43. 10.1016/j.neuroimage.2014.06.012 24945670

[56] Allen PJ Fish DR Smith SJ. Very high-frequency rhythmic activity during SEEG suppression in frontal lobe epilepsy. Electroencephalogr Clin Neurophysiol. (1992) 82:155–9. 10.1016/0013-4694(92)90160-J 1370786

[57] Fisher RS Webber WR Lesser RP Arroyo S Uematsu S. High-frequency EEG activity at the start of seizures. J Clin Neurophysiol. (1992) 9:441–8. 10.1097/00004691-199207010-00012 1517412

[58] Lee SA Spencer DD Spencer SS. Intracranial EEG seizure-onset patterns in neocortical epilepsy. Epilepsia. (2000) 41:297–307. 10.1111/j.1528-1157.2000.tb00159.x 10714401

[59] Grenier F Timofeev I Steriade M. Neocortical very fast oscillations (ripples, 80-200 Hz) during seizures: intracellular correlates. J Neurophysiol. (2003) 89:841–52. 10.1152/jn.00420.2002 12574462

